# From plots to commercial fields: scalable, transferable cotton morphology and productivity estimation using functional growth proxies from UAV and PlanetScope time series

**DOI:** 10.1016/j.plaphe.2026.100220

**Published:** 2026-05-08

**Authors:** Francesca Devoto, Michael Bange, Carlos Camino, William Woodgate, Scott Chapman, Andries Potgieter

**Affiliations:** a Queensland Alliance for Agriculture and Food Innovation, The University of Queensland, Brisbane, Australia; b Cotton Seed Distributors Ltd, Wee Waa, NSW, Australia; c Lab. Geo-information Science and Remote Sensing. Wageningen University, Netherlands; d School of the Environment, The University of Queensland, 4072, QLD, Australia; e CSIRO, Space and Astronomy, Kensington, 6151, WA, Australia; f Terrestrial Ecosystem Research Network, The University of Queensland, 4072, QLD, Australia; g School of Agriculture and Food Sustainability, The University of Queensland, Brisbane, Australia

**Keywords:** Operational and scalable sensing framework, Remote sensing growth proxies, Multispectral UAV and high-resolution satellite imaging, Single and multi-date growth dynamic metrics

## Abstract

Product demand and climate variability are progressively increasing the need for real-time, scalable crop monitoring to support varietal selection and in-season input optimisation. However, producers still have limited information on the temporal and spatial variability of cotton health and performance beyond point-scale field surveying. In addition, given cotton's high phenotypic plasticity, near real-time derived metrics are essential to improve input efficiency and strengthen long-term sustainability of the cotton industry in Australia. Therefore, we proposed a functional integrated predictive sensing framework to estimate and predict cotton canopy morphological (i.e., height) and productivity traits (i.e., dry matter and lint yield) across large plots (12m × 6m). Scalability was validated by applying the proposed framework to estimate and map cotton yield across commercial fields. To do this, we explored the accuracy of high-resolution multispectral imagery from two platforms (unmanned aerial vehicle (UAV) and PlanetScope (PS)) collected across two 144-plot trials for two cotton seasons. These were designed with a large range in nitrogen rates (N), shading, and two growth-regulator doses, thus, creating variable environments. Sensing metrics were obtained from UAV imagery (1.3–1.6 cm pixel size) acquired once in 2022/23 and eight times in 2023/24, while PS composites (3 m pixel size) provided near-daily coverage in both seasons. Time-series gaps were imputed using Savitzky–Golay smoothing in thermal time (GDD), enabling extraction of growth dynamic metrics (GDMs) as single-date (SD; e.g., peak canopy) and multi-date (MD; e.g., daily average growth rate) metrics. After reducing collinearity and dimensionality, random forest (RF), support vector regression (SVR), and gaussian process regression (GPR) were trained and interpreted with SHAP, for feature contribution. UAV single-date models (SD_ UAV) achieved strong accuracy for height (R^2^ = 0.77), biomass (R^2^ = 0.73), and yield (R^2^ = 0.81). Incorporating UAV time-series metrics (MD_UAV) improved the performance R^2^ = 0.87, 0.86, and 0.85 for height, biomass and yield, respectively. Application of the derived models using high resolution satellite data (MD_PS) for different farming systems showed highly significant accuracy (R^2^ = 0.67) to predict cotton yield at aggregated field scale. As such, enabling the detailed spatial prediction of cotton yield within a field. It is anticipated that the proposed functional sensing framework will improve the estimation of key cotton production traits, supporting field- and within-field decision-making, ultimately contributing to more resilient and sustainable cotton production in Australia.

## 1 Introduction

Cotton (*Gossypium spp.*) is a perennial shrub cultivated as an annual crop across tropical and subtropical latitudes in more than 80 countries, with China, India, and the United States among the leading producers [1]. While Australia contributes only 3–5% of global production, its cotton is internationally recognised for quality, environmental stewardship, and innovation [2]. This reputation is supported by strong research frameworks, including the myBMP program, and guided by the industry's Vision 2029 [3]. However, climate change, water scarcity, soil degradation, and biodiversity loss continue to threaten sustainable growth and efficiency in cotton production. Addressing these challenges requires improved tools for monitoring crop health mainly focussing on traits like height (canopy morphology) and production (e.g. biomass and yield) in near real-time. Thus, understanding the crop status and input responsiveness is necessary to support location specific decisions for better informed agronomic management.

Previous research on cotton has shown that monitoring of specific crop traits (e.g., number of mainstem nodes, height and leaf area index, ‘LAI’) can provide critical insights into canopy growth dynamics, supporting input optimisation, and enhancing productivity [4,5]. For example, optimal vegetative growth before flowering is critical for boll development [6]. Hence, resource limitations trigger “cutout” [7], when new vegetative and reproductive growth cease, while excess resources induce “rank growth,” favouring vegetative over reproductive structures [8]. Such phenotypic plasticity generates substantial variation in height, biomass, and yield across environments and management regimes. Plant height, often measured along with the node number, reflects the environmental conditions and stress. Hence, the height-to-node ratio (HNR) is a useful stress indicator [9]. While node development is mainly temperature-driven, stresses typically affect internode length, making plant height more responsive than node number. Indeed, stresses such as delayed irrigation shorten internodes without strongly affecting node formation [10]. In addition, biomass is a key trait, integrating phenology and photosynthetic activity at the plant and canopy level. However, most of these traits (i.e., counting of nodes and bolls (fruits), leaf area, biomass and height) are currently collected manually at small number of locations (2 to 3) in a field. Monitoring can be labour intensive and, because of this, opportunities to account for field variation are limited and biases exist when small numbers of samples are taken [11]. Additionally, unlike many cereal crops where final yield is strongly associated with biomass and LAI, cotton's perennial habit makes it highly sensitive to disruptions in carbohydrate partitioning [12]. This further complicates cotton growth and production monitoring. For example, environmental stresses such as heat or insect pressure can induce heavy fruit shedding, shifting resources to vegetative growth and delaying reproductive development, ultimately reducing yield [13].

Remote sensing (RS) offers scalable, non-destructive tools to monitor crop within field variability (e.g., yield and biomass in wheat, reaching R^2^ = 0.9 [14]), track phenology (e.g., in barley and wheat [15]), and forecast yield (e.g., in wheat, up to R^2^ = 0.93 [16]) in near real time. However, trait estimation in cotton remains challenging due to its complex canopy structure, asynchronous flowering, and overlapping vegetative and reproductive phases [17]. Recent advances in UAV (unmanned aerial vehicle) and high-resolution satellite platforms are a promising alternative to manually collated field data. Furthermore, they offer readily available high spatial, spectral, and temporal resolution imagery, to monitor and estimate canopy health and production traits. Combined with machine learning (ML), which can model complex non-linear relationships, RS data has demonstrated utility to predict morphological, biochemical, and physiological features at field scales [18]. In addition, UAV-based sensing with RGB or multispectral sensors has shown strong potential for estimating height, canopy structure, and biomass in cotton. Hence, by mathematically combining the spectral bands into equations it is possible to build vegetation indices (VIs). These have emerged as valuable tools for exploring plant properties non-invasively, being proxies for morphological (incorporating the near-infrared (NIR) spectral region or band [19]) and biochemical features (involving the red edge (RE) spectral region). Traditional indices such as the normalized difference vegetation index (NDVI) have been widely used to track crop biophysical attributes non-destructively and at high temporal frequency. For example, NDVI has shown significantly high correlations of R^2^ = 0.87 and 0.96 with cotton structural parameters LAI, height, respectively [4]. In this sense, Pokhrel, Virk [20] demonstrated that lint yield in cotton is primarily governed by the fraction of light intercepted by the canopy (IPAR), radiation use efficiency (RUE), and harvest, emphasising the importance of canopy dynamics during early growth stages. However, as outlined by Adams, Ritchie [21], the NDVI tends to saturate at values around 0.8 [22], generally corresponding to LAI of about 3 in cotton [23].

Despite these advances, most cotton RS studies rely on limited data captured at a few dates during the season (e.g., squaring, first flower, boll filling), which restricts their ability to capture temporal dynamics of canopy growth. Hence, the accuracy in estimating canopy traits from above varies throughout the growing season, generally peaking during vegetative and early reproductive phases. Thus, the timing of the image acquisition is critical, as the model performance is highly dependent on the crop growth stage [24]. Recent work has shown that incorporating temporal features improves trait estimation. For example, da Silva Andrea, de Oliveira Nascimento [25] observed that adding days after emergence (DAE) as predictor in ML models enhanced the accuracy of canopy height estimation from PlanetScope (PS) satellite imagery. Cotton's perennial growth habit and sensitivity to the interactions between the genotypes with environmental conditions and under management practices (G × E × M) make it particularly suited to time-series approaches that track growth patterns [26]. As such, time-series RS data also enables the reconstruction of growth trajectories, integrating the cumulative effects of E and M throughout the season. Time-integrated VIs have been shown to correlate strongly with lint yield, reflecting their potential as proxies for photosynthetic capacity and biomass [27]. Consequently, beyond observations related to a specific timepoint in the growing season (i.e., single-date, SD, such as peak canopy), curve-fitting of VIs provides growth metrics (e.g., the VI's rate of change over a given interval), covering a period of time (i.e. multi-date, MD) which are directly linked to yield [28]. These curve-derived features, including the area under the curve (AUC) and the daily average growth rate (GR), have already improved yield prediction in other crops, underlining their importance for monitoring seasonal dynamics [29,30]. Furthermore, plant development is a physiological process primarily driven by the accumulation of heat, not the passage of time [31]. Therefore, estimating growth using phenological derived sensing metrics against growing degree days (GDD), rather than calendar days or days after sowing (DAS), strengthens the representation of temperature-driven processes and supports cross-site generalisation [32]. Early knowledge of crop growth traits such as plant height, biomass accumulation, growth rate, and canopy cover provides cotton farmers critical opportunities to make timely, data-driven management decisions. Yield potential is largely established early in the season through fruit retention before cutout, while later growth stages primarily protect this potential through boll filling and fibre quality [33].

This work directly spawned from a recent industry workshop where the need was highlighted for more accurate, non-destructive, and scalable monitoring tools that can deliver timely information across large production areas.

Therefore, the main aim of this study was to determine whether remotely sensed growth dynamic metrics (GDMs) derived from reconstructed growth curves, from multispectral UAV and PS imagery, on GDD can be used as proxies to accurately predict key cotton morphological (i.e., height) and productivity (i.e., aboveground biomass and lint yield) traits, providing an operational, industry-ready framework. The specific objectives were to:

(i) Quantify the relationships between UAV multispectral GDMs and cotton morphological and productivity traits at single (SD) and across multiple (MD) time periods;
(ii) Evaluate the scalability of UAV-based approaches to satellite sensing for predicting canopy height and yield across large plot scales; and
(iii) Validate the scalability and application of the proposed functional sensing framework to commercial fields for predicting yield across spatial scales.

## 2 Materials and methods

### 2.1 Study site

**Fig. 1:**
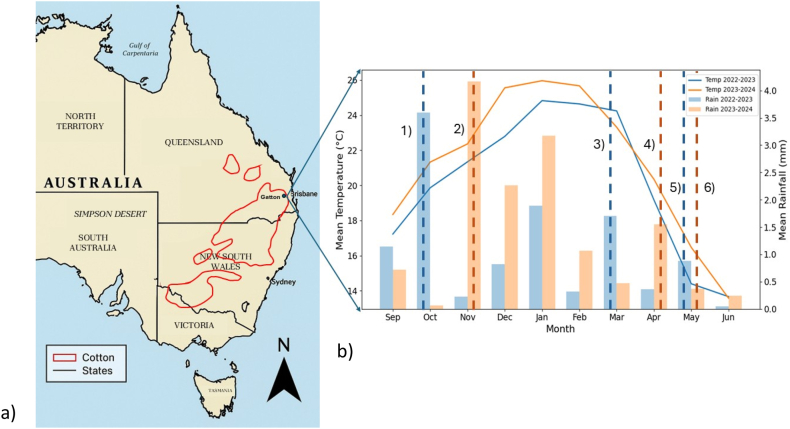
(a): Australia with states boundaries (black) and the broad cotton growing region (red), adapted from source [37]. (b): Monthly average temperature (line chart) and total average rainfall (bar chart) for the 2022/23 (blue) and the 2023/24 (orange) summer cotton growing seasons recorded at UQ Gatton campus (Sourced from Ref. [38]). In addition, the vertical stippled lines (blue 22/23 and red 2023/24) depict sowing dates (1 and 2), biomass cut dates (3-4) and the harvest dates (5-6), for each season, respectively.

The field experiment was conducted over two consecutive summer growing seasons (2022/23 and 2023/24) at the University of Queensland's Gatton Campus (152°19′54″ E, 27°34′1″ S), in southeast Queensland, Australia (Fig. 1) [34]. Observations of crop traits (i.e., canopy height, dry matter and yield) and sensing data (i.e., multispectral UAV images) were collected as part of an existing cotton management trial (more details in section 2.2). The region has a subtropical climate, with summer temperatures ranging, on average, between a minimum of 19 °C and a maximum of 32 °C, and an average of 5 to 8 rainy days per month. Monthly precipitation ranges from 41 mm to 109 mm [35]. The soil is classified as a black Vertosol [36], with approximately 60% clay content within 90 cm profile, deep cracking and self-mulching properties, alkaline pH (8.0–8.6), total N levels below 0.17%, and nitrate-N levels below 3 mg kg^−1^ in the 0–90 cm soil layer. To investigate the scalability of the proposed framework, Cotton Seed Distributors Ltd. (CSD; industry partner) provided eight commercial fields (30–60 ha) for the 2024–25 season, in southeast Queensland.

### 2.2 Experimental data design for variability in cotton canopy traits and commercial fields

The cotton management experiment was established to assess canopy and yield responses under contrasting environmental and management conditions using a single genotype (Sicot 748B3F). Released in 2015–16, this cultivar has insect traits (B3F; Bollgard 3 that provides three different modes of action to target Helicoverpa species) and herbicide traits (Roundup Ready Flex which includes resistance to over-the-top applications of glyphosate) [39]. Sowing occurred on 7 November (2022/23) and 7 December (2023/24) at a density of 13 seeds m^−1^ to achieve a stand of 10 plants m^−1^. Each trial comprised 144 plots (12 m × 6 m; 1 m row spacing; appendix 9.1, Figure 7), arranged in three and six replicates, respectively. Treatment variability was introduced through different nitrogen (N) rates and timings, shading, and, in 2022/23 only, high growth regulator applications.

Eight N rates, ranging from 0 to 350 kg/ha in 50 kg/ha increments were applied. The N fertiliser, an urease inhibitor (Green Urea NV® 46% N, Incitec Pivot Fertilisers, VIC, Australia), was applied using two strategies: either as a single pre-sowing application or as a split application, with half applied pre-sowing and the other half during early squaring (in 2023, at 64 DAS; 447 GDD) or flowering (in 2024, 69 DAS; 700 GDD).

Shading cloths reducing incoming radiation by 30% were used to modulate photosynthetic energy availability and influence boll retention and yield [40]. In 2022/23, cloths were installed three times at 10-day intervals from first flower (79 DAS, 572 GDD) to cut-out (136 DAS, 1085 GDD); in 2023/24, twice from 815 GDD (81 DAS) to 1066 GDD (110 DAS). Shading was applied to plots receiving 0, 50, 150, 250, and 350 kg N ha^−1^, with upfront N in 2022/23 and split N in 2023/24.

Mepiquat chloride (MQ) was applied to both N strategies to restrict growth and enhance fruit retention by altering carbon allocation. Three yield potential scenarios were tested: (1) standard MQ rate, (2) triple MQ rate, and (3) standard MQ combined with shading [41,42]. Three yield potential scenarios were established: (1) a standard application of MQ, (2) an application of approximately three times the standard rate, and (3) a combination of shading with the standard MQ rate. A total of ≈105 ml L^−1^ MQ was applied across treatments at squaring (72 DAS, 514 GDD; 30 ml L^−1^) and early boll-filling (112 DAS, 865 GDD; 75 ml L^−1^); high-rate plots received additional applications at first flower (79 DAS, 572 GDD; 100 ml ha^−1^) and early boll-filling (112 DAS, 865 GDD; 75 ml ha^−1^). Cotton was irrigated via an overhead sprinkler system supplying 345 mm of water per season at two to four week intervals, from two weeks after sowing until maturity. Weeds, diseases, and pests were managed following standard agronomic practices [43,44] and defoliation was performed according to the *Australian Cotton Production Manual* [45].

The commercial validation fields spanned substantial genetic (G), environmental (E), and management (M) diversity: multiple cultivars were grown within and across sites, fields were distributed across eight locations (∼100 km apart) in southern Queensland, and management differed mainly in water supply (six irrigated and two rainfed fields). Together, this G × E × M variability generated a broad range of growing conditions.

### 2.3 Observed traits and sensing data

#### 2.3.1 Morphological and production traits

Canopy height (m) and dry matter (Mg/ha) were determined from biomass cuts conducted in plots receiving 0, 50, 150, 250, and 350 kg N/ha, totalling 70 and 90 plots out of 144, for the first and the second season, respectively. Sampling took place on 22–23 March 2023 (136 DAS; 1085 GDD) and 11–12 April 2024 (127 DAS; 1187 GDD), just prior to crop defoliation to capture peak biomass conditions (Table 1). From each plot, plants were harvested within a 2 m^2^ (2022/23) and 1 m^2^ (2023/24) area on two rows (appendix 9.1, Figure 7). In non-shaded plots, samples were collected from rows two and five, at 2 m from the plot edge. In shaded plots, the region of interest (ROI) included rows three and four, positioned 1 m inside the shade cloth. During the 2022/23 season, other four cotton plants, lying just next to each end of the 2 × 1 m biomass-cut rows, were sampled to assess plant growth parameters (e.g. plant height, number of nodes). In contrast, during the 2023/24 season the samples were used for the plant mapping, including detailed counting of node development, average plant height, boll number, vegetative and fruiting branching counts, and recording of total fruit retention. Canopy height was measured with a tape measure and calculated as the average plant height per plot. Following this, the plant material was oven-dried at 60 °C to determine aboveground biomass carbon content (C%), dry matter (Mg/ha), N uptake (kg/ha, calculated as the product of biomass N concentration and dry matter), and N concentration (N%). After drying, samples were weighed and pulverised for N analysis. Finally, mechanical harvesting of rows three and four from all 144 plots occurred on 31 May 2023 (203 DAS; 1273 GDD) and 28 May 2024 (137 DAS; 1305 GDD), following defoliation. Seed cotton yield and gin turnout were recorded, enabling the calculation of lint yield and seed N removal. For the season 2024-25 with the eight commercial fields, the final yield was provided by CSD for each site and variety.

**Table 1:** Morphological (i.e., height (m)) and productivity (dry matter (Mg/ha) and yield (kg/ha)) canopy traits collected on multiple plots (n) on different dates and related days after sowing (DAS), growing degree days (GDD) and phenological stages across two seasons.

Season	Variables	Traits		
		Morphological	Productivity	
		Height (m)	Dry matter (Mg/ha)	Yield (kg/ha)

2022-2023	n	70		144
	Date	22-23 March		30 May
	DAS	136		204
	GDD	1085		1273
	Stage	Full mature – before defoliant		Harvest

2023-2024	n	90		144
	Date	11-12 April		28 May
	DAS	127		137
	GDD	1187		1305
	Stage	Full mature – before defoliant		Harvest

#### 2.3.2 Multispectral data onboard a UAV

For the 2022/23 season, a single UAV flight was conducted on 28 February (113 DAS; 883 GDD) near peak canopy development using a MicaSense Altum camera. Imagery was acquired at a fixed altitude of 30 m, providing a ground sampling distance (GSD) of 1.34 cm per pixel for the multispectral bands and 20 cm per pixel for the thermal band (Table 2).

**Table 2:** UAV flights in both cotton seasons. The number of observed plots (first number) is followed by the ground resolution (xy) in cm and the altitude of flight (h) in m. Each flight date is related to the respective phenological stage from vegetative one (V) to flowering (F), four to eight nodes above the first position white flower (NAWF) until harvest (H). Additionally, the respective growing degree days (GDD) and days after sowing (DAS) are specified.

Season	UAV	Date								
		January		February			March	April		May
		4	22	7	14	28	15	2	15	23
2022/23	Plots (xy, h)					144 (1.8, 40)				
	Stage					Cutout and full canopy				
	GDD					883				
	DAS					113				

2023/24	Plots (xy, h)	144 (1.35, 30)	144 (1.35, 30)	144 (1.8, 40)	144 (1.8, 40)		144 (1.6, 35)	144 (1.6, 35)	144 (1.6, 35)	144 (1.6, 35)
	Stage	V (6-7 nodes)	V (14-16 nodes)	F (18-19 nodes & 8 NAWF)	4-8 NAWF		Cutout and full canopy	Fully mature		H
	GDD	276	467	642	709		991	1121	1204	1306
	DAS	29	47	63	70		100	118	131	169

During the 2023/24 season, data were collected using a MicaSense AltumPT camera across eight dates covering the full cotton growth cycle (MicaSense Inc., Seattle, USA; https://support.micasense.com/hc/en-us). Table 2 summarises the UAV campaigns, including flight dates, number of plots imaged, flight altitude (h), and corresponding spatial resolutions. Flights were conducted by AirBorn Insight Pty Ltd (https://airborninsight.com.au/) at regular intervals from 4 January (emergence) to 23 March 2024 (pre-harvest). Depending on h, the spatial resolution ranged from 1.3 to 1.8 cm for multispectral imagery and from 8.5 to 11.3 cm for thermal one.

The Altum and AltumPT sensors capture six spectral bands (Figure 8, appendix 9.2): blue (B, 400–500 nm; 32 nm bandwidth), green (G, 500–600 nm; 27 nm), red (R, 600–680 nm; 14 nm in Altum, 16 nm in AltumPT), red edge (RE, 680–750 nm; 12 nm), near-infrared (NIR, 750–1050 nm; 57 nm), and long-wave infrared (LWIR, 7500–13,500 nm; 6 μm). The AltumPT additionally includes a panchromatic band to enhance spatial detail.

For each flight, four ground control points (GCPs) were placed at the corners of the experimental area and georeferenced using Propeller Aeropoints (Propeller, Australia). Images were collected with 80% front and side overlap and acquired within 2 h of solar noon to minimise variation in illumination. Radiometric calibration was performed using nadir images of a MicaSense reflectance calibration panel captured before and after each flight. To ensure consistency across dates, flights were completed within 15–20 min under clear or minimally cloudy conditions between 10:00 and 12:00 local time.

#### 2.3.3 PlanetScope satellite data

PS ortho analytic surface reflectance imagery was obtained through the education and research (E&R) program [46]. Level-3B products with eight spectral (Figure 8, appendix 9.2) narrow bands were acquired: coastal blue (431–452 nm, 20 nm bandwidth), blue (B, 465–515 nm, 50 nm bandwidth), green I (GI, 513–549 nm, 36 nm bandwidth), green II (GII, 547–583 nm, 36 nm bandwidth), yellow (Y, 600–620 nm, 20 nm bandwidth), red (R, 650–680 nm, 31 nm bandwidth), red edge (RE, 697–713 nm, 15 nm bandwidth), and near-infrared (NIR, 845–885 nm, 40 nm bandwidth). The imagery provides a ground sampling distance of 3 × 3 m, near-daily revisit frequency, and is projected onto a standard cartographic reference system.

### 2.4 Analytical framework

**Fig. 2:**
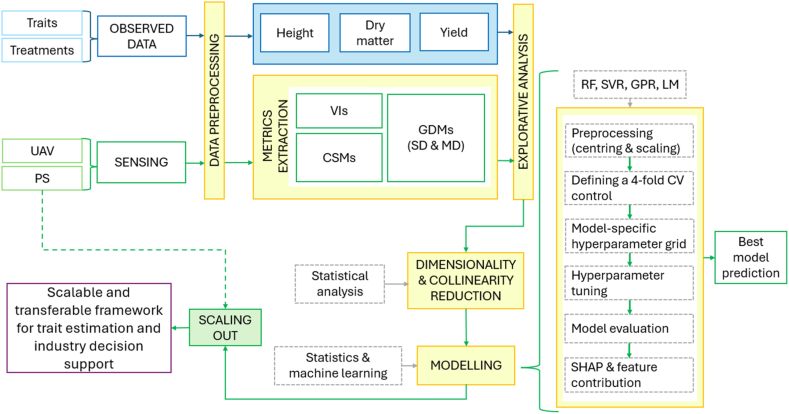
Workflow for predicting cotton traits from sensing-derived metrics. Ground-truth observations (height, dry matter, yield) were integrated with UAV and PlanetScope (PS) imagery to extract vegetation indices (VIs), canopy structural metrics (CSMs), and growth-dynamic metrics (GDMs). After dimensionality and collinearity reduction, statistical and machine learning models (random forest (RF), support vector regression (SVR), gaussian process regression (GPR) and linear model (LM)) were applied to predict the morphological and productivity traits. The best-performing models were then scaled out using PS imagery to predict yield at field scale.

To predict cotton morphological and productivity traits, in-field cotton canopy observations were integrated with RS data from UAV and PS platforms across plots and seasons by following the steps depicted in Fig. 2: data collection and preprocessing, metrics extraction, explorative analysis, dimensionality and collinearity reduction, modelling and scaling out. Ground-truth measurements of plant traits (i.e., height, dry matter, and lint yield), together with experimental treatment information, were collected concurrently with UAV and PS imagery acquisitions.

All datasets underwent a preprocessing pipeline: UAV imagery was mosaiced and georeferenced, and PS imagery was rescaled to spectral reflectance. Subsequently, plot-level metrics were extracted, including VIs, canopy structure metrics (CSMs, section 2.6.1) from the 3D pointcloud, and GDMs derived from SD and MD imagery (refer to section 2.7.3 for more details). To characterise temporal dynamics, VI time series were interpolated and fitted with growth curves, from which GR, AUC, and SD values corresponding to key phenological stages (e.g., maximum GR, peak canopy, biomass sampling) were derived.

The extracted metrics were subjected to explorative analysis, including dimensionality reduction and collinearity assessment via principal component analysis (PCA) and Pearson correlation [47], to identify the most informative and minimally correlated variables. These selected metrics were then used in predictive modelling, calibrated and validated against ground-truth observations. Model interpretability was assessed using SHAP (SHapley Additive exPlanations) values to quantify the contribution of each metric to trait prediction.

Finally, the validated models were scaled out using PS satellite data, beyond the plot-level analyses, producing continuous estimates of cotton growth and productivity, thereby demonstrating the potential for broader adoption across varied industry applications.

#### 2.4.1 Approaches for growth proxies

**Fig. 3:**
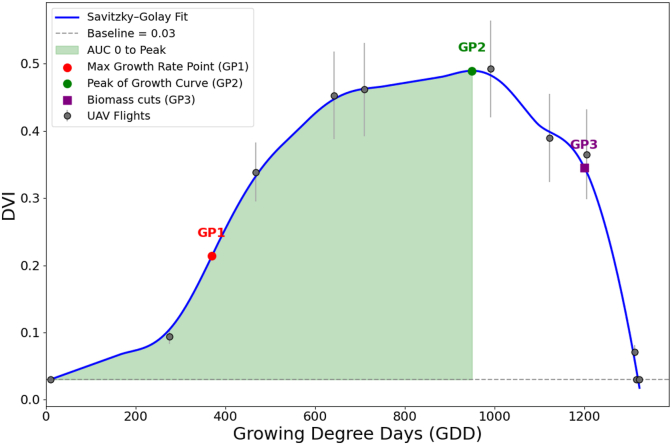
Reconstructed difference vegetation index (DVI) curve from UAV data averaged across plots. Grey dots represent individual flights (± standard deviation). Growth phases (GPs) were identified as: GP_1_, maximum growth rate (red); GP_2_, peak canopy (green); and GP_3_, biomass cut (purple). The green area represents the AUC from sowing to GP2, and the dashed grey line indicates the soil baseline value.

Trait predictions were evaluated across three key growth phases (GPs) that reflect different stages of crop development, extracted from the growth curve for both UAV and satellite data. GP_1_ (early growth) corresponded to the maximum GR period, when canopy expansion was rapid, but biomass accumulation was still limited. GP_2_ (peak canopy) represented the stage of maximum canopy cover, where VIs typically stabilised and provided the strongest spectral signal. GP_3_ (biomass accumulation) reflected later growth, when the canopy had reached maturity and structural biomass dominated trait expression (Fig. 3). By comparing predictions across these stages, the analysis captured both early detection potential (GP_1_) and the more stable predictive relationships evident at peak canopy and biomass maturity (GP_2_ and GP_3_).

Three approaches were employed for trait prediction (Table 3), which varied by season, data source, GP, and predictor variables.

• SD_UAV: A SD approach using the peak canopy UAV multispectral images collected in both 2022/23 and 2023/24 seasons, to estimate canopy height, dry matter, and yield. This approach relied on SD morphological and biochemical VIs, with ROIs defined at the whole-plot level (165 plots for height and dry matter; 288 plots for yield) and two seasons' data.
• MD_UAV: A one season MD time-series analysis from UAV multispectral imagery throughout the 2023/24, to model canopy height, dry matter, and yield (144 samples). Eight UAV image dates were combined with ground destructive measurements (biomass cuts and yield samples) across the three GPs. Predictors included both SD and MD VIs, biochemical variables from images collected at GPs 1–3, and CSMs derived from DEMs.
• MD_PS: Growth-curve reconstruction using PS multispectral composites from the 2022/23 and 2023/24 seasons to predict canopy height (95th percentile as proxy). Predictors included SD and MD indices, and VIs capturing biochemical traits (including RE information). Pure vegetation pixels were selected and aggregated by N treatment following section 2.6.2 and supplementary 1.1.2-1.1.3, resulting in 16 aggregated points across the two seasons.
• Yield scalability assessment: A total of 125 plots across two seasons were used for training ML, using GDMs for SD and MD and biochemical VIs from PS. The best-performing model was then transferred to eight commercial fields in the 2024/25 season (section 2.6.3) to generate yield estimates at both field scale and within-field spatial variability.

**Table 3:** Overview of the three approaches employed to predict cotton canopy traits, differing in season, scale, data source, growth periods (GPs), predictor variables (i.e., single and multi-date, SD and MD, biochemical (Bio) and canopy structure metrics, CSMs), and regions of interest (ROIs).

Approach (Traits)	Scale	Season	Data	GP	Predictors	Description	ROI (samples)
SD_UAV (Height, dry matter, yield)	Large plot	2022/23 2023/24	• One multispectral UAV image • Height, dry matter, yield	2	SD and Bio	Images at GP_2_	Whole plot (165 for height and dry matter; 288 for yield)

MD_UAV (Height, dry matter, yield)	Large plot	2023/24	• Eight multispectral UAV images • Height, dry matter, yield	1	SD and MD	GDMs to GP_1_	• Biomass cuts (90) • Yield (144)
				2	SD and MD	GDMs to GP_2_	
					Bio	Image at GP_1_ and GP_2_	
					CSMs	DEM at GP_2_	
				3	SD and MD	GDMs to GP_3_	
					Bio	Image at GP_1_, GP_2_ and GP_3_	
					CSMs	DEM at GP_3_	

MD_PS (Height)	Large plot	2022/23 2023/24	• PS multispectral composites • Height (95 Percentile), yield (calibration)	1	SD and MD	GDMs to GP_1_	• Pure pixels (16) • Whole plot average (95 percentile)
				2	SD and MD	GDMs to GP_2_	
					Bio	closest to the GP	
MD_PS (Yield scalability)	Large plot	2022/23 2023/24	• PS multispectral composites • Yield (calibration)	1	SD and MD	GDMs to GP_1_	• Pure pixels & yield (125)
				2	SD and MD	GDMs to GP_2_	
					Bio	closest to the GP	
	Field	2024/25	• PS multispectral composites • Yield (scale out)	1	Same predictors used in calibration		• Pure pixels & yield (8)
				2	Same predictors of the calibration		

The primary distinction between these approaches lies in the GP or timepoint considered. Each GP incorporated all metrics available up to that stage, integrating both historical and stage-specific data. Three GPs were defined:

• GP_1_: sowing to the day of maximum GR.
• GP_2_: sowing to peak canopy.
• GP_3_: sowing to the day of the biomass cuts.

**Fig. 4:**
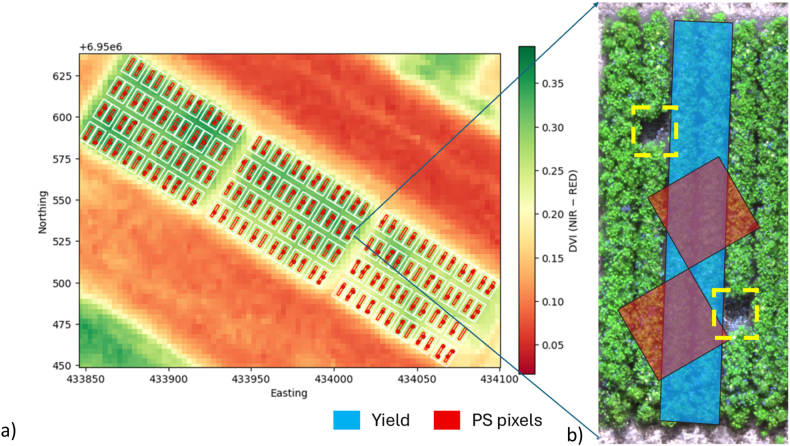
(a) DVI maps for the cotton trial (season 2023-2024) from PlanetScope (PS). The white polygons are the plots boundaries, while the red ones are the inward buffering. The red dots highlight the pure vegetated pixels extracted for each plot. (b) Zoom on a plot with the biomass cuts (yellow dashed squares), the yield ROI for harvest (blue) and the fully enclosed PS pixels (red).

Across approaches, predictors included morphological features (from SD and MD metrics), biochemical VIs for all traits, and CSMs specifically for height. The ROI differed according to each approach (Fig. 4 and appendix 9.1, Figure 7).

Together, these complementary strategies captured different levels of temporal detail and spatial scalability: SD_UAV provided rapid, single-time insights; MD_UAV integrated temporal, biochemical, and structural information; and MD_PS offered scalable satellite-based monitoring aligned with UAV campaigns.

### 2.5 Preprocessing of imagery

#### 2.5.1 UAV multispectral imagery orthomosaics

Following the methodology described by Smith, Chen [48], the Pix4D Mapper Pro software (version 4.7.5) was used to generate point clouds for georeferencing and stitching raw UAV images into orthomosaics, as well as for radiometric calibration and the creation of DEMs.

Pix4D's dedicated reflectance map generation engine enabled radiometric calibration. This was performed by interpolating of the relationship between the reflectance values of the nadir image of a MicaSense calibration panel and the timestamps of the images captured before and after each flight. This approach allowed in-flight reflectance values to be corrected based on estimated reflectance at specific time points. The average ground control point (GCP) marker error across the orthomosaics was 2 cm.

#### 2.5.2 Management of satellite data

The eight band Planet data product was downloaded using an end-to-end Python-based workflow. The Planet API (https://www.planet.com/) user dashboard was used to specifying a minimum of 80% cloud-free cover over the entire experimental area and all cloud free pixels were used, no additional filter data were applied. The product was already radiometrically calibrated and atmospherically corrected through Planet's processing pipeline [49].

### 2.6 Metrics extraction

#### 2.6.1 UAV imagery metrics

ROIs were manually digitized in QGIS (https://qgis.org/) to ensuring alignment of the in-field geometry where manual measurements were taken for all dates and seasons (appendix 9.1, Figure 7; appendix 9.3, Table 5).

The SD_UAV adopted the whole plot as ROI to extract the zonal statistics for VIs for both seasons, since both peak images were collected before the cuts. The MD_UAV employed the ROIs for the biomass cuts and yield during the season 2023/24. For biomass cuts, the sampled area was 1 m^2^. However, the ROI shapefile was drawn to cover 3 m^2^ (3 m × 1 m), extending 1 m before and after the cut zone. This adjustment allowed a larger area more representative for the plot, without compromising the information (see supplementary section 1.1.1). Yield ROIs were defined as 24 m^2^ (12 m × 2 m). This corresponded to the two middle rows, harvested in each plot.

The feature extraction was performed for each ROI and flight date using Xtractori [50], a Python-based tool. This computed plot-scale zonal statistics for a suite of morphological and biochemical VIs, as well as CSMs features, summarised in table 5 (appendix 9.3) along with the spectral bands and equations. The NDVI [51] was excluded due to known saturation effects occurring at values near 0.7–0.8 in cotton canopies. Among the CSMs, the canopy volume and the 95th percentile of the DEM were extracted. Canopy volume was estimated as the sum of pixel-level heights multiplied by the corresponding pixel area. This provides an aggregate measure of canopy volume above ground level. The 95th percentile height metric was calculated by sorting all pixel height values and selecting the value below which 95% of the measurements falls, offering a robust indicator of typical canopy height.

#### 2.6.2 Accounting for mixed pixels using satellite data at the plot scale

To account for soil/background effects in PS, we retained only fully vegetated pixels within each plot using an inward buffer (Fig. 4 and supplementary section 1.1.2). VIs (Table 5 in appendix 9.3) were computed from these pixels and averaged at plot level, then min–max normalized to further reduce residual background and bidirectional reflectance effects [52] (supplementary section 1.1.3). Because biomass cut sampling areas (dashed yellow squares, Fig. 4) were small and not well aligned with the pure-vegetation pixels (red squares, Fig. 4), MD_PS was limited to canopy height (using the 95th percentile as a proxy) and yield. The 95th percentile, extracted from peak canopy images in both 2022/23 and 2023/24, was averaged per plot. The remaining between-plot variability was mitigated by aggregating by N-rate treatments [53,54]. Yield prediction was calibrated at plot level using average VIs per plot for both seasons and then scaled out to the eight commercial fields provided by CSD for the 2024/25; to maximise calibration sample size, no N-rate aggregation was applied for the yield model. This allowed to more accurately scale the approach out to commercial fields using satellite data.

#### 2.6.3 Spatial assessment of background effects for field scale application

A 20 m inward buffer was applied to the eight commercial fields to reduce edge effects, using the same method described in section 2.6.2 for the plot-level analysis. Mean VI values were then computed across each buffered field to produce only one value for each field and date.

### 2.7 Reconstructing of daily crop growth profiles

#### 2.7.1 Gap-filling approach for crop growth dynamics

To capture crop dynamics, time series were reconstructed into continuous growth profiles by testing multiple curve fitting approaches, as shown in our previous research [55]. For each plot was chosen a Savitzky-Golay (SG) filter approach [56,57], on all morphological VIs (Table 5, appendix 9.3), as more stable curve-fitting technique for this dataset. Originally developed by Savitzky and Golay [58], the SG filter is a computational technique designed to smooth experimental data by reducing random noise while preserving meaningful signal features. It is particularly suited for numerical datasets such as spectral measurements or time series of VIs [57]. After the pre-processing steps on the images and alignment to GDD, the same the SG was applied according to the methodology described in the supplementary 1.1.4 to both UAV and PS VIs.

#### 2.7.2 GDD (thermal time) for phenological development

GDD provide a thermal-time scale that tracks crop phenology (stage progression) rather than calendar days. Fitting growth curves to accumulated GDD normalises for temperature differences across sites and seasons, aligns key crop growth curves from Vis with exact phenology stages (e.g., emergence, flowering, maturity), at daily timescales. Structural VIs were plotted against accumulated GDD computed using Equation (1) [59] as follow:

Eq. [1] $$GDD=\left\{ \begin{array}{c} \frac{\left(\text{Tmax}+\text{Tmin}\right)}{2}-15.6DD\\ \frac{\left(32+\text{Tmin}\right)}{2}-15.6DD \end{array}\right.$$

where Tmax and Tmin are the daily maximum and minimum temperatures, respectively. When Tmax exceeds 32 °C, it is restricted at 32 °C to reflect the species' optimal development threshold. Any negative DD values are set to zero, ensuring no accumulation on cold days. Daily maximum and minimum temperatures required for this computation were sourced from the nearby weather station [38].

#### 2.7.3 Growth curve dynamic metrics

From the reconstructed crop growth profile, we derived GDMs, for both SD and MD approaches for UAV and PS imagery (Fig. 3). SD metrics captured the value of each VI at specific GPs (e.g., the day of maximum GR (GP_1_), peak canopy (GP_2_), and the biomass cut date (GP_3_)). GP_1_ was identified as the maximum of the first derivative, GP_2_ as the highest VI observed during the season, and GP_3_ as the VI value on the biomass cut day. MD metrics summarised periods of the curve and included daily GR and the AUC from sowing to each GP, providing a phenological basis for modelling cotton growth and yield. For UAV data, GR was computed as the average VI change per GDD from sowing to GP_1_ and GP_2_, and AUC was obtained via numerical integration from sowing (0 GDD) to each GP (Table 5, appendix 9.3). The same framework was applied to PS time series, with additional SD and MD metrics extracted up to 400 and 800 GDD to capture squaring and maximum boll size, respectively.

### 2.8 Dimensionality and collinearity reduction

Including too many input variables often fails to improve model performance and can increase overfitting. To reduce data complexity and collinearity, we applied principal component analysis (PCA) across all three approaches (SD_UAV, MD_UAV, and MD_PS) in RStudio (v4.5.1) (Fig. 2) [60]. For interpretability, we used Varimax rotation to maintain orthogonality and simplify the loading structure [61]. Each variable was assigned to the principal component (PC) on which it had the highest absolute loading, and only variables with Varimax-rotated loadings >0.15 were retained. We then computed Pearson correlations to remove near-duplicate information: predictors showing very high intercorrelations (|r| > 0.95) with other variables, while also having low correlation with the dependent variable, were excluded. The remaining predictors, capturing morphological (SD and MD), biochemical, and CSMs information, formed the final input set for the modelling pipeline.

### 2.9 Exploratory analysis approach

#### 2.9.1 Feature contribution by SHAP

To quantify the marginal contribution of each sensing metric to canopy trait prediction within the ML models (Fig. 2), we applied SHAP as a post-hoc, model-agnostic method. Based on cooperative game theory, SHAP assigns each feature a value representing its contribution to individual predictions relative to a baseline (the model's mean prediction from a background dataset) [62,63]. Feature importance was summarised using the mean absolute SHAP value (mean|SHAP|), with larger values indicating stronger influence on model outputs. Further details on SHAP visualisation and interpretation are provided in supplementary 1.1.5.

#### 2.9.2 Machine learning model implementation

Three ML approaches (i.e, random forest (RF), support vector regression (SVR), and gaussian process regression (GPR)) were trained to predict the dependent variables in the SD_UAV, MD_UAV and MD_PS (only for yield) datasets (Fig. 2). Predictor sets varied by approach, GP, and trait, and included morphological (e.g., SD, MD), biochemical, and CSMs. Model tuning used repeated four-fold cross-validation (CV) with a 75:25 training–testing split and a leave-one-out repetition scheme (n–1) in RStudio (v4.4.4). Hyperparameters were optimised by grid search [64], selecting configurations that minimised out-of-fold root mean squared error (RMSE). More details about model settings and optimised hyperparameters are provided in supplementary section 1.1.6. For the MD_PS approach on height, a simple linear model (LM) was applied between the 95th percentile and the most explanatory sensing metrics, as the aggregation by N-rate produced 16 samples in total.

#### 2.9.3 Model evaluation

The model's accuracies were determined with coefficient of determination (R^2^) [65] and the RMSE by pooling all out-of-fold predictions across the n-1 CV iterations, ensuring unbiased estimates of model generalisation performance.

## 3 Results

### 3.1 Dimensionality and collinearity reduction across approaches

PCA-based dimensionality reduction and Pearson correlation filtering were used to identify informative, independent predictors across datasets, temporal scales, and approaches, reducing redundancy among VIs, CSMs, and GDMs. PCA consistently explained >94% of the variance, and correlations with observed traits were strong (r > 0.8 for most variables), supporting their predictive relevance. As a result, the initial predictor sets (5–82 variables) were reduced to small, non-redundant subsets (typically 2–6 variables per trait and growth stage), improving interpretability and model stability. Retained features, including EVI, OSAVI, DVI, GEMI (SD and MD), NDRE, and canopy height percentile, captured complementary morphological, biochemical, and temporal information. Full details on variable counts, selected predictors, loadings, variance explained, and trait correlations are provided in appendix 9.4.

### 3.2 Single date estimates of trait using UAV data

#### 3.2.1 Feature contributions of VIs to model accuracy

**Fig. 5:**
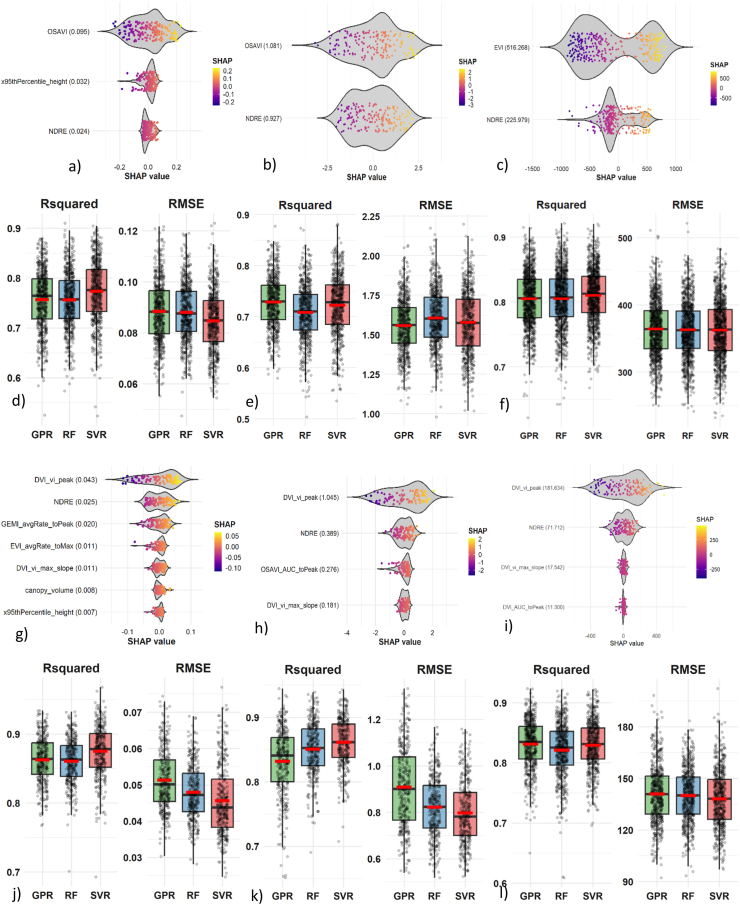
Violin plots for the SD_UAV (a-c) and MD_UAV at GP_2_ (g-i) illustrate SHAP value distributions of SVR, as best-performing model in both approaches for height (a, g), dry matter (b, h) and yield (c, i). Features are ranked in decrescent order by mean|SHAP| (in brackets), representing their contribution to the model. This is expressed in m for height (a, g), Mg/ha for dry matter (b, h) and in kg/ha for yield (c, i). The samples are coloured by SHAP values (viridis scale, while the violin widths represent value density). Boxplots show out-of-fold R^2^ and RMSE for GPR (green), RF (blue), and SVR (red). Boxes represent the 25th–75th percentiles, with medians (black lines), means (red crossbars), whiskers (1.5 × IQR), and individual resample values (black dots). The results are related to the SD_UAV (d-f) and MD_UAV at GP_2_ (j-l).

SHAP analysis quantified the marginal contributions of indices across models (Fig. 5 a-c). For canopy height, OSAVI had the greatest effect in SVR and GPR, shifting predictions by 0.09 m and 0.06 m, respectively. In contrast, NDRE, was most influential in RF (mean|SHAP| = 0.06). For dry matter, OSAVI again contributed more strongly than NDRE in SVR (1.54 Mg/ha) and GPR (1.19 Mg/ha), while NDRE dominated in RF (mean|SHAP| = 1.17 Mg/ha). For yield, EVI was the leading predictor across models, with mean|SHAP| values ranging from 457.7 to 511 kg/ha, for exceeding those for NDRE (195.5-228.3 kg/ha).

#### 3.2.2 Model calibration and performance

All three algorithms produced statistically significant results (p < 0.01), with comparable accuracies across traits (Fig. 5 d-f). Specifically, canopy height (165 samples) was estimated with R^2^ > 0.77 and RMSE = 0.08 m, with SVR showing a slight advantage over the other models. For dry matter (165 samples), accuracies were R^2^ > 0.73 and RMSE <1.61 Mg/ha, with GPR performing marginally better in comparison to the other models. Yield (288 samples) was predicted with R^2^ = 0.81 and RMSE = 363 kg/ha, where SVR had a small outperformance. Full model results across traits and algorithms are presented in appendix 9.5 for height, dry matter and yield.

### 3.3 Predicting canopy traits from sequential multi-spectral UAV data

#### 3.3.1 Most informative variables from SHAP

The DVI, as a SD metric both at the maximum GR, used in GP_1_, and at peak canopy, included in GP_2_ and GP_3_, consistently emerged as the most influential predictor across traits and models. At GP_1_, SVR was the best-performing algorithm, DVI achieved mean|SHAP| values of 0.10 m for height, 1.9 Mg/ha for dry matter, and 233 kg/ha for yield, reflecting the average magnitude of shifted predictions.

At later stages (GP_2_), NDRE closely followed DVI across all models. From SVR (Fig. 5 g-i), DVI and NDRE shifted predictions by 0.042 m and 0.027 m for height, 1.04 and 0.39 Mg/ha for dry matter, and 229 and 54 kg/ha for yield, respectively.

At GP_3_, DVI showed mean|SHAP| values of 0.04 m for height, 1.2 Mg/ha for dry matter, and 221 kg/ha for yield, while NDRE contributed 0.02 m, 0.45 Mg/ha, and 56 kg/ha, respectively. Consistent patterns of feature importance were observed across all other ML algorithms (data not shown).

#### 3.3.2 Model accuracies of height, dry matter and yield throughout phenological stages

Height (90 samples) was predicted with statistical significance and moderate to high accuracy across all GPs and models. At GP_1_, SVR outperformed the other approaches, achieving R^2^ of 0.75 (RMSE = 0.06 m), compared with R^2^ of 0.68 (RMSE = 0.07 m) for GPR and 0.73 (RMSE = 0.06 m) for RF. Higher accuracies were observed at GP_2_ (Fig. 5j) and GP_3_, with comparable performance across models and a slight advantage for SVR, which showed R^2^ = 0.88 (RMSE = 0.05 m) at GP_2_ and R^2^ = 0.87 (RMSE = 0.04 m) at GP_3_. Both GPR and RF consistently achieved R^2^ = 0.86 (RMSE = 0.05 m) across these stages.

Dry matter (90 samples) predictions followed a similar trend, with lower accuracies early in the season (GP_1_) and higher accuracies at later stages (GP_2_ and GP_3_). At GP_1_, GPR achieved a slight advantage over the other models with R^2^ = 0.74 and RMSE = 1.52 Mg/ha. At GP_2_ (Fig. 5k) and GP_3_, prediction accuracies improved, with SVR showing a small outperformance, reaching R^2^ = 0.86 and RMSE = 0.79 Mg/ha across both GPs.

Yield (144 plots) confirmed the overall pattern, with lower accuracies at GP_1_ compared to GP_2_ and GP_3_, where results stabilised. At GP_1_, SVR slightly outperformed the other models, with R^2^ = 0.72 and RMSE = 178 kg/ha. At GP_2_ (Fig. 5l) and GP_3_, SVR again showed a slight advantage, reaching R^2^ values of 0.83 and 0.85 with corresponding RMSEs of 138 and 131 kg/ha.

Tables in appendix 9.5 summarise the results for height, dry matter, and yield across approaches, GPs, and all ML models.

### 3.4 Satellite imagery for canopy height and yield estimation at early and peak canopy stages

#### 3.4.1 Height estimation using the percentile height and PS imagery

Height prediction was statistically significant across both growth periods (p < 0.001), but performance was lower in GP_1_ (DVI at 400 GDD: R^2^ = 0.40; RMSE = 0.07 m) than in GP_2_, where accuracy improved markedly (EVI at peak canopy: R^2^ = 0.76; RMSE = 0.04 m; Table 4).

**Table 4:** Prediction performance (R^2^) for each trait across locations and seasons at two growth periods (GP1 and GP_2_). Significance is indicated as ∗ p < 0.05, ∗∗p < 0.01, and ∗∗∗p < 0.001. Canopy height was modelled with a linear model (LM), whereas yield was best predicted using random forest (RF). For percentile height, predictors were DVI at 400 GDD (DVI_400GDD) in GP1 and EVI at peak canopy (EVI_peak) in GP_2_. For yield, GP1 used DVI at maximum growth rate (DVI_maxGR), CLRE from the PlanetScope image closest to maximum growth rate (CLRE_maxGR), and DVI AUC from sowing to maximum growth rate (DVI_AUC_to_maxGR); GP_2_ used DVI at peak canopy (DVI_peak), CLRE at peak (CLRE_peak), and DVI AUC from sowing to peak canopy (DVI_AUC_to_peak). The same predictors were used for field-scale scaling-out, yielding statistically significant results in GP_2_ only (GP1 not significant).

Height estimation				
Trait	Location (samples: season)	ML	GP_1_	GP_2_
			DVI_400GDD	EVI_peak

Percentile height	Large plot (16: 2022/23-2023/24)	LM	0.4∗∗	0.75∗∗∗

**Scaling out for field scale decisions**				
Trait	Location (samples: season)	ML	DVI_maxGR + CLRE_maxGR + DVI AUC_maxGR	DVI_peak + CLRE_peak + DVI AUC_peak

Lint yield	Calibration - Large plot (164: 2022/23-2023/24)	RF	0.48∗∗	0.72∗∗
	Scaling - Fields (8: 2024/25)	RF	//	0.67∗

#### 3.4.2 Scalability of the proposed framework for field scale decisions

For yield (Table 4), CV at the large-plot scale used a combination of morphological and GDMs for both GPs, including DVI at maximum GR (GP_1_) and at peak canopy (GP_2_), DVI-based AUC (from sowing to maximum GR (GP_1_) and to peak canopy (GP_2_)), and CLRE from the PS images closest to GP_1_ and GP_2_. This delivered moderate-to-high accuracies in both periods (GP_1_: R^2^ > 0.48, RMSE <538 kg/ha; GP_2_: R^2^ > 0.72, RMSE <491 kg/ha).

This calibration step was followed by the testing at field scale, producing statistically significant performance only for GP_2_ (Table 4, RF: R^2^ = 0.67; RMSE = 1044 kg/ha).

A concise summary of height and yield results is provided in appendix 9.5.

## 4 Discussion

### 4.1 Trait prediction from UAV imagery: single-date vs. multi-date approaches

Cotton trait monitoring matters because the crop grows indeterminately, developing vegetative tissues and reproductive organs simultaneously. Management therefore focuses on balancing canopy “source” capacity with “sink” demand from new branches and bolls to maximise yield and fibre quality. Reliable estimates of traits such as height, biomass, and yield support real-time decisions on nitrogen and irrigation while limiting environmental impacts. When collected with UAVs or satellites, these measurements also reduce field labour and give breeders scalable data to select high-yielding, stress-resilient genotypes.

The SD_UAV approach explained over 75% of trait variability with strong statistical significance, indicating that key traits can be estimated from a single UAV image captured at peak canopy. This can lower field and flight costs because peak canopy is a critical window when LAI and photosynthetic area are near maximum and canopy condition is closely tied to yield formation [66]. Peak canopy typically occurs around, or soon after, cutout (≈5 NAWF, nodes above the first position white flower), when boll demand matches or exceeds photosynthetic supply and growth shifts from producing new nodes to filling bolls [67]. However, the timing is highly environment-dependent: stress (e.g., drought or low N) can trigger premature cutout and an earlier, smaller peak, while excess resources or fruit shedding can delay cutout and extend canopy expansion. As a result, the method can provide useful information about the peak canopy, plant heath and stress, but it is highly sensitive to image timing, does not capture growth dynamics, and offers limited insights once the crop has already reached its peak growth.

Conversely, MD_UAV increased peak-canopy prediction accuracy for canopy traits by ∼10%, likely because it provides ML models with richer information: time-series growth patterns and GDM that integrate SD and MD features with biochemical proxies and crop status measures, capturing temporal changes in chlorophyll and LAI that are physiologically linked to yield formation. Hence, early-season GR (green-up slope) captures vigour and input responsiveness; stress (e.g., N deficiency) typically flattens this trajectory, limiting peak canopy and hastening senescence. Curve inflection points can also mark when vegetative expansion slows as reproductive demand rises, signalling a shift in source–sink balance. Additionally, fitting curves in thermal time (GDD) strengthens biological interpretability by aligning phenological transitions across seasons, environments, and sowing dates better than calendar time. GDD-derived metrics, such as AUC, summarise both the intensity and duration of canopy greenness (“greenness-days”), acting as proxies for cumulative photosynthetic capacity and, therefore, lint yield. This explains why MD_UAV can outperform the SD_UAV approach and remain informative across the season. Importantly, MD_UAV enabled earlier prediction (∼38 DAS) than previously reported [68]. Prediction accuracy increased by >10% from early season (GP_1_) to peak canopy (GP_2_) and then stabilised from GP_2_ onward (see section 3.3 for GP_2_–GP_3_). This pattern aligns with Gutierrez, Norton [22], who reported weaker performance during early growth than at peak canopy, likely because low early-season biomass and LAI limit the ability of spectral indices to represent photosynthetic capacity and yield potential. Consistent with this, peak bloom was the most suitable stage for lint-yield estimation, when canopy development is sufficient and closely linked to yield components [22].

Therefore, depending on the specific purpose and location, either an SD or MD approach can be used. However, it should be recognised that the SD approach loses more information related to the crop growth and typically results in lower prediction accuracy compared to MD.

### 4.2 Height prediction using high-resolution PlanetScope at large plot scales

The MD_PS approach demonstrates the scalability and operational potential rather than absolute accuracy. As already suggested in section 2.6.2 and in the supplementary 1.1.2-1.1.3, the coarser spatial resolution of PS (3 m) introduces mixed pixels and consequently lower accuracy in trait estimation compared with UAV-based data. Nevertheless, the accuracies were still statistically significant across traits and growth stages, following a similar trend to UAV-based approaches. Hence, accuracies improved later in the season, by up to 30% (e.g., yield, R^2^ = 0.76 to 0.91), in comparison to early stages.

### 4.3 Moving towards a predictive phenotyping framework to support cotton industry in Australia

#### 4.3.1 Scaling out of sensing metrics for yield estimation at commercial fields scales

The MD_PS yield application at field scale demonstrates that sensing metrics from high-resolution satellite imagery can effectively support the scaling-out of key agronomic traits for industry use (section 3.4). Because the fields were planted with commercial cultivars, some shared across sites, the dominant drivers of variability were expected to be management (e.g., irrigated vs rainfed) and environment (sites ∼100 km apart), rather than genotype, supporting the potential for broader application. However, as shown in Figure 9 (appendix 9.6), the model tends to overpredict yield when transferred from the large-plot training scale to commercial fields. This may partly reflect the narrower N range at field scale compared with the plot trial, leading to different growth and yield variability. In addition, field-scale aggregation effectively averaged yields across cultivars; although cultivar performance was broadly comparable within each site, accuracy could likely improve if the provided cultivar distribution and spatial yield information are available, to enable stratified pixel aggregation. Overall, the proposed framework enables near real-time, within-field estimation of RS-derived variables and metrics to support proactive agronomic decisions under biotic and abiotic stresses.

Moreover, two practical constraints commonly affect RS sensing workflows: (i) environmental conditions that limit data availability (e.g., cloud cover) and (ii) processing demands associated with large image time series. In this study, we addressed these challenges by implementing a robust gap-filling strategy to handle missing or noisy UAV and PS observations, enabling the extraction of targeted, biologically meaningful sensing metrics at any phenological stage. Looking ahead, continued advances in Earth observation data streams and AI-driven processing are expected to further streamline model calibration and deployment at field scale across diverse G × E × M conditions [69].

#### 4.3.2 Scalability and transferability implications for operational decision making in the Australian cotton industry

In response to industry demand, timely, non-destructive, and scalable cotton monitoring across large production systems, are needed for improved agronomical and strategical decision-making at field and regional scales. As such, this study presents an integrated framework to estimate labour-intensive field data related to cotton yield with earth observation (EO) data, driven by a robust and scalable ML approach.

**Fig. 6:**
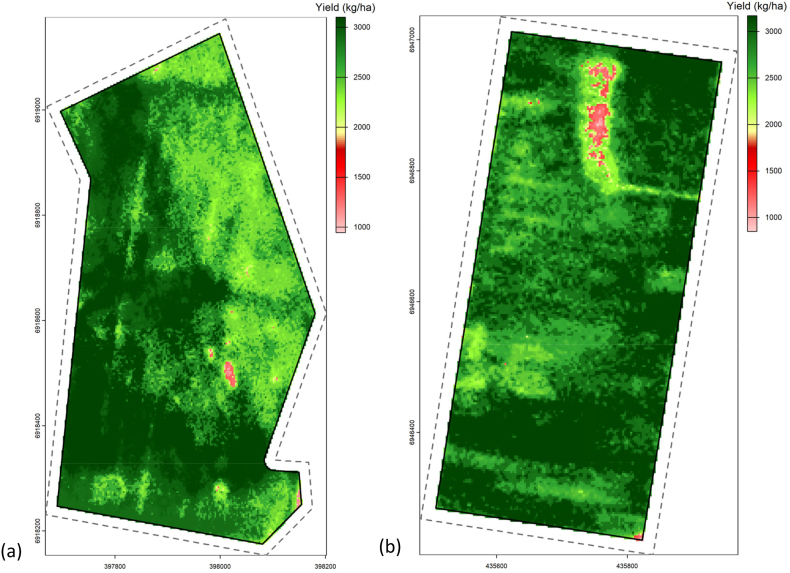
Yield maps (R^2^ = 0.72, RMSE = 491 kg/ha, p-value <0.01) for (a) Nobby dryland field (60ha) and (b) Forest Hill irrigated (20 ha). The colour represents the estimated yield from red (low) to green (high). Yield can vary within large fields, due to, for example, soil-driven effects (e.g., slope, panel a) and irregular irrigation performance (e.g., drip lines with blocked emitters, panel b). These constraints can cause stunted growth in some zones and more vigorous growth in others, generating pronounced within-field spatial variability, which can be assessed by site-specific agronomic decisions.

**Fig. 7:**
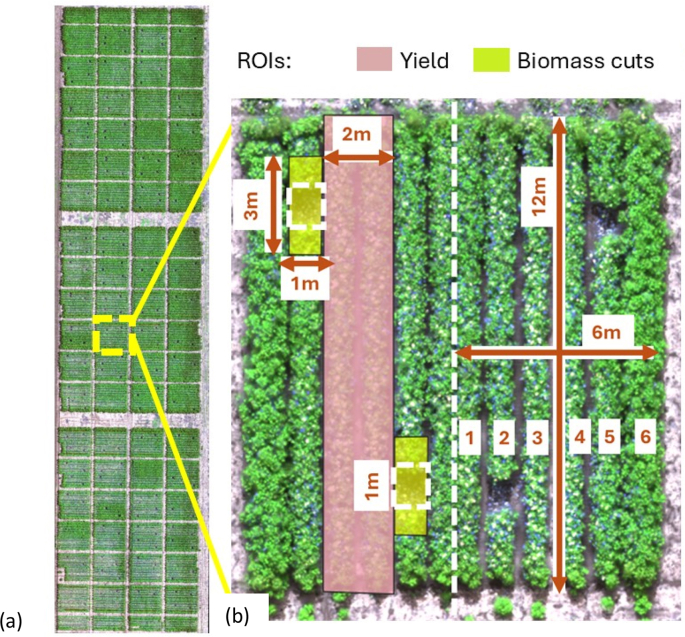
(a-b) Experimental layout and the regions of interest (ROIs) used for trait measurement. Panel (a) shows the full trial layout consisting of 144 plots captured by UAV imagery on 15 April 2024, immediately after the biomass cuts (highlighted by the white dashed square). Panel (b) provides a zoomed view of two representative plots (12 m × 6 m each, separated by a white dashed vertical line) with six rows per plot. The ROIs used for destructive sampling are highlighted: yellow squares (3 m × 1 m) for biomass cuts and pink rectangles (12 m × 2 m) for yield.

**Fig. 8:**
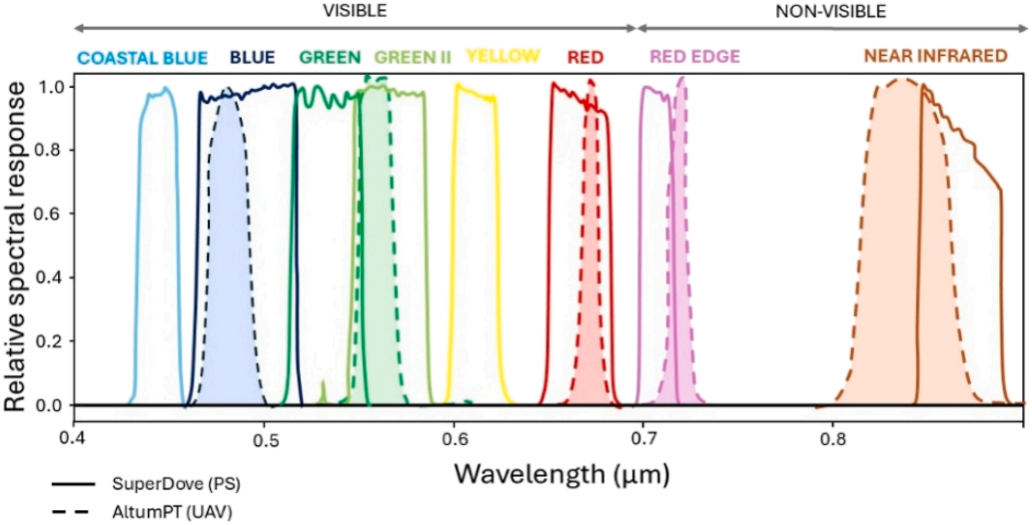
Relative spectral response from visible to near-infrared (NIR) of PlanetScope (PS) SuperDove (eight bands, continuous line) and AltumPT (five bands, dashed line and colour filled channel areas). Adapted from: [73,74].

**Fig. 9:**
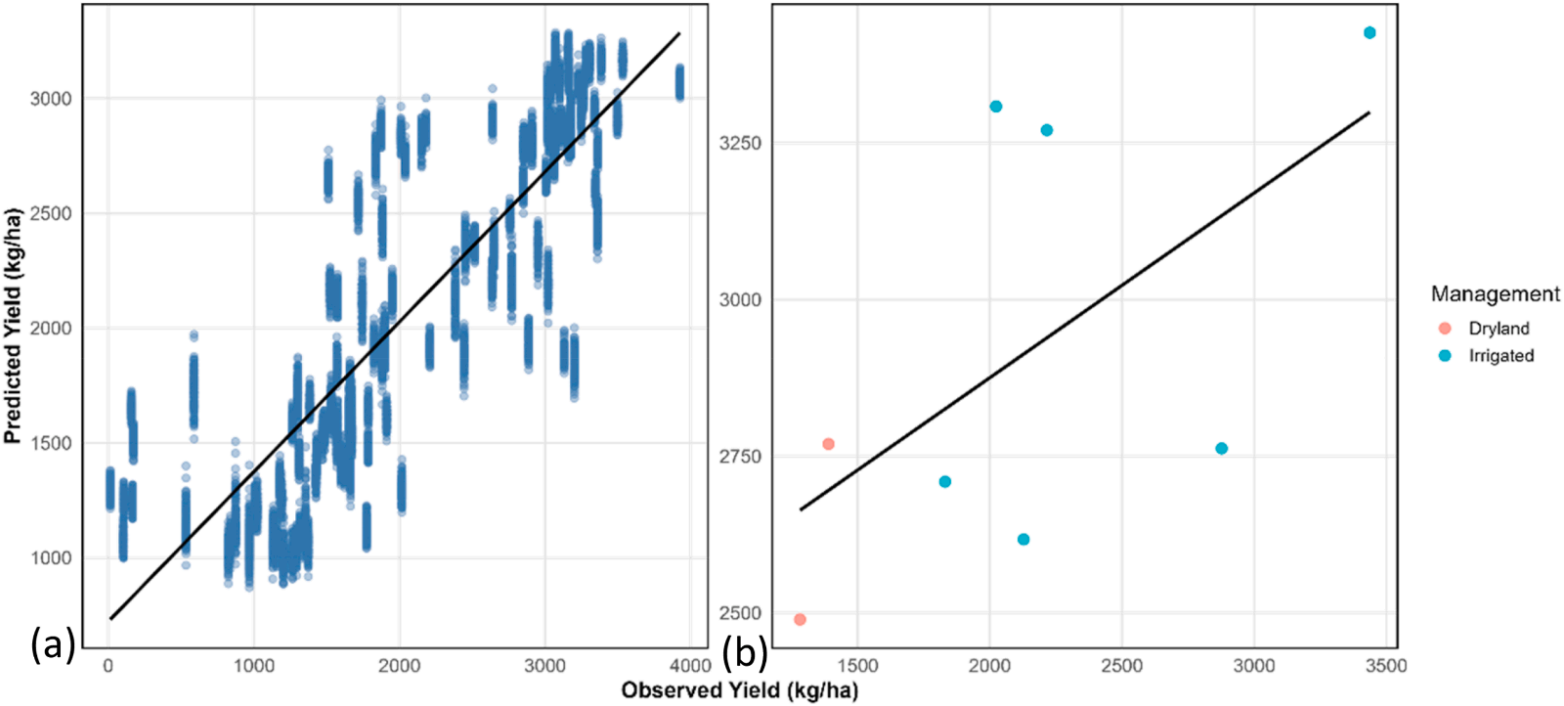
Scatter plot of observed versus predicted lint yield from (a) cross-validation (4-fold CV, 124 repeats: R^2^ = 0.72, RMSE = 491 kg/ha, p-value <0.01) and (b) test at the field level (eight sites: R^2^ = 0.67, RMSE = 1044 kg/ha, p-value = 0.01) of the random forest model.

Scalability was assessed by applying the best-performing model across eight commercial cotton fields (30–60 ha) provided by Cotton Seed Distributors Ltd. (CSD). Calibration under well-managed, irrigated conditions across two seasons (144 plots × two years) and eight N rates helped isolate predictive signals from confounding stress factors. Additionally, using the widely adopted cultivar Sicot 748B3F strengthened the robustness of the framework, providing a conservative baseline for broader application. The sites also captured a wide range of genotype × environment × management (G × E × M) conditions. Hence, these included multiple cultivars, irrigated and dryland systems, different row configurations, and geographically distinct locations (∼100 km apart), ensuring robust evaluation under real-farm variability. As a result, the EO–ML model achieved moderate to high predictive accuracy, explaining 48–72% of yield variability at both field and within-field scales (Fig. 6).

It is anticipated that outputs from the proposed EO-ML framework will be particularly relevant in Australian cotton systems. Hence, the cotton strong phenotypic plasticity and large farm sizes make routine ground monitoring impractical. At the same time, precise management of nitrogen, irrigation, and growth regulators is critical to ensure both profitability and sustainability. Thus, spatial estimates of crop traits related to health and stress will better inform producers on in-season agronomic decisions regarding variable-rate input applications. Specifically, early-season indicators of canopy development (e.g., GP_1_, early growth at inflection point) can inform fertiliser and irrigation scheduling [70,71], while post-flowering metrics (e.g., GP_2_, as peak canopy) can guide growth regulator applications to control excessive vegetative growth and protect yield potential [72].

To show transferability, we reconstructed growth curves using temperature-driven GDD. As such, allowing EO-ML derived features to remain site-specific and align consistently with the actual plant development. This improved comparability across seasons, environments, and management practices. Therefore, the site-specific traits were reliably predicted by the dynamic integration of near-daily EO and temperature (GDD) data throughout the growth period, confirming the transferability of the methods.

Finally, this study clearly demonstrated that functionally derived traits from UAV and high-resolution satellite data show moderate to high utility and accuracy for scaling cotton production and morphological attributes (including yield) across a range of agronomic management strategies at temporal and spatial scales. As such, providing a strong scientific foundation for deploying EO–ML approaches at field, farm and regional scales.

## 5 Conclusion

This study shows that high-resolution RS can reliably capture key cotton canopy growth traits from large plot to field scales, providing a simple, implementable framework for both industry decision support and phenotyping. By calibrating a unified sensing–ML pipeline across UAV and PS data over two seasons and fitting growth curves in thermal time (GDD), we improved generalisability and demonstrated that informative SD and MD metrics can be extracted throughout the season. From ∼82 candidate predictors per trait spanning temporal dynamics, CSMs, and biochemical proxies, we identified key descriptors (e.g., OSAVI/EVI for SD_UAV; DVI/NDRE for MD_UAV; EVI- and GEMI-based growth metrics for MD_PS) for the measured traits (R^2^ ≈ 0.75–0.90). Combining SD and MD features consistently increased accuracy (>10%) relative to SD alone and enabled the trait prediction early in the season (e.g., from 38 DAS, with about 75% of variance explained), highlighting the value of extracting meaningful curve-based information. Importantly, these GDMs have clear biological meaning: early green-up and GR descriptors reflect vigour and stress responsiveness, inflection points relate to shifts in vegetative–reproductive balance, and GDD-based AUC summarises cumulative canopy “greenness-days” (a proxy for seasonal photosynthetic capacity and yield potential). Therefore, our findings highlight the utility of sensing-ML-derived metrics for early and non-destructive assessment of canopy traits, emphasising the importance of tracking intermediate variables such as biomass accumulation and canopy height alongside final yield. This study also sets out the research steps needed for robustly exploring the relationships between morphological, biochemical and canopy structural sensing metrics to canopy traits like yield and height. Applying a functional framework, as proposed here, to large and diverse commercial fields demonstrated the extended scale to farmer fields for future operational deployment. Extending this approach to additional cotton-production environments is expected to better characterise temporal and spatial yield variability at field scale, enabling more proactive, site-specific agronomic decisions (e.g., fertiliser and growth-regulator management), thus leading more sustainable cotton production in Australia.

## Author contributions

**F. Devoto**: Conceptualization, Methodology, Software, Validation, Formal Analysis, Investigation, Writing-original draft, Visualisation. **M. Bange**: Conceptualization, Review & Editing, Supervision. **C. Camino**: Review & Editing. **W. Woodgate**: Review & Editing, Supervision. **S. Chapman**: Review & Editing, Supervision. **A. Potgieter**: Conceptualization, Methodology, Writing - Review & Editing, Supervision, Visualisation, Funding acquisition.

## Declaration of generative AI and AI-assisted technologies in the manuscript preparation process

During the preparation of this work the authors used ChatGTP and ClaudeAI in order to support coding and polish grammar of the manuscript. After using these tools, the authors reviewed and edited the content as needed and take full responsibility for the content of the published article.

## Declaration of competing interest

The authors declare that they have no known competing financial interests or personal relationships that could have appeared to influence the work reported in this paper.

## Figures and Tables

**Table 5:** Summary of vegetation indices (VIs), canopy structure metrics (CSMs), and growth dynamic metrics (GDMs) derived from multispectral imagery. VIs represent morphological (M) and biochemical (B) traits, CSMs describe canopy structure, and GDMs include single-date (SD) and multi-date (MD) features, such as values at maximum growth rate (GRmax), peak canopy, biomass cut, daily growth rate (GR), and area under the curve (AUC). For PlanetScope (PS) data, SD and MD metrics were also extracted at 400 and 800 growing degree days (GDD).

Feature	Metric		Equation	Ref.

M	DVI		R_800_ − R_670_	[75]
	EVI		2.5 × [(R_800_ − R_670_)/(R_800_ + 6R_670_ − 7.5R_400_ + 1)]	[76]
	GEMI		∗η × (1 − 0.25 × η) – [(R_670_ − 0.125)/(1 − R_670_)] η = [2 (R_800_^2^ − R_670_^2^) + 1.5 R_800_+ 0.5 R_670_]/(R_800_ + R_670_ + 0.52)	[77]
	OSAVI		1.16 × (R_800_ − R_670_)/(R_800_ + R_670_ + 0.16)	[78]

B	Cl_RE_		(R_800_/R_710_) − 1	[79]
	NDRE		(R_800_ + R_710_)/(R_800_ − R_710_)	[80]

CSMs	Canopy volume		$\frac{\sum_{i=1}^{n}h_{i}*GSD}{Area\;of\;ROI}$	[81]
	95th Percentile		$h_{1}\leq h_{2}\leq \ldots \leq h_{n}$ k=[0.95∗n] $P=H_{k}$	[82]

GDMs	SD	GR_max_ (GP_1_)	$\max\left(\frac{\Delta VI}{\Delta GDD}\right)$	
		400GDD (only for PS)	VI(GDD_400_)	
		800GDD (only for PS)	VI(GDD_800_)	
		Peak (GP_2_)	max(VI)	
		Biomass (GP_3_)	VI(GDD_biomass_)	
	MD	GR • 0- GP_1_ • 0-400GDD (only PS) • 0-800GDD (only PS) • 0- GP_2_	$\frac{\Delta VI}{\Delta GDD}$	
		AUC • 0- GP_1_ • 0-400GDD (only PS) • 0-800GDD (only PS) • 0- GP_2_ • 0- GP_3_	$\int_{0}^{GP}f\left(GDD\right)d\left(GDD\right)$	

**Table 6:** SD_UAV number (n.) of principal components (PCs) explaining a given percentage (%) of variance, for each trait at peak canopy (GP_2_). For each trait, the number of initial (first number) and retained (in brackets) variables after the dimensionality and collinearity steps are specified. The retained variables are shown with the respective loadings within each PC and correlation coefficient (r) with the dependent variable (Trait).

Trait	GP	Initial (retained) variables	Retained variables	PCs (%)	Loadings (PC1; PC2; PC3)	r
Height	2	6 (3)	OSAVI	3 (96)	0.25, 0.36, 0.14	0.84
			NDRE		0.37, 0.19, 0.21	0.84
			x95thPercentile_height		NA, −0.18, 0.9	0.63
Dry matter		5 (2)	NDRE	2 (94)	0.51	0.83
			OSAVI		0.4; −0.24	0.84
Yield		5 (2)	NDRE	2 (94)	0.2; −0.5	0.7
			EVI		0.35; −0.32	0.86

**Table 7:** Summary of input and retained variables for the MD_UAV approach across growth phases (GPs) for each trait. The table reports the number of principal components (PCs), variance explained (%), loading values, and correlation coefficients (r) with the dependent crop trait. Retained variables include single-date (SD) metrics—index values at maximum growth rate (max_slope), peak canopy (peak), and biomass cut (at_BiomassCuts)—and multi-date (MD) metrics, such as daily average growth rate (avgRate) and area under the curve (AUC). Both biochemical and canopy structural metrics were considered.

Trait	GP	Initial (retained) variables	Retained variables	PCs (%)	Loadings (PC1; PC2; PC3)	r

Height	1	8 (2)	EVI_avgRate_toMax	2 (98)	0.253; 0.421	0.71
			DVI_vi_max_slope		0.352	0.86
	2	21 (6)	DVI_vi_peak	3 (97)	0.316	0.93
			NDRE		0.177; NA; −0.325	0.93
			GEMI_avgRate_toPeak		0.287	0.9
			EVI_avgRate_toMax		NA; 0.448	0.71
			DVI_vi_max_slope		0.256	0.71
			canopy_volume		−0.482	0.87
			95thPercentile_height		−0.165; 0.124; −0.586	0.79
	3	24 (5)	DVI_vi_peak	3 (97)	0.12; NA; −0.263	0.93
			NDRE		0.312	0.93
			GEMI_avgRate_toPeak		0.113; NA; −0.23	0.9
			EVI_vi_at_BiomassCuts		0.271; NA; −0.105	0.92
			DVI_vi_max_slope		NA; 0.259	0.86
			canopy_volume		0.372; 0.121; 0.226	0.87

Dry matter	1	8 (2)	DVI_vi_max_slope	2 (97)	0.432	0.81
			DVI_AUC_toMaxSlope		0.147; 0.351	0.6
	2	10 (4)	DVI_vi_peak	3 (97)	0.257; NA; −0.17	0.93
			NDRE		0.467; NA; 0.22	0.9
			OSAVI_AUC_toPeak		0.303	0.89
			DVI_vi_max_slope		0.396	0.81
	3	18 (3)	DVI_vi_peak	2 (97)	0.218; −0.108	0.93
			DVI_vi_max_slope		−0.342	0.81
			NDRE		0.325	0.9

Yield	1	8 (2)	DVI_vi_max_slope	2 (98)	0.337; 0.125	0.8
			DVI_AUC_toMaxSlope		0.117; 0.403	0.64
	2	18 (4)	DVI_vi_peak	2 (97)	0.284	0.91
			NDRE		0.394; 0.193	0.9
			DVI_AUC_toPeak		0.218; −0.111	0.8
			DVI_vi_max_slope		−0.427	0.8
	3	18 (3)	DVI_vi_peak	3 (97)	0.221	0.91
			NDRE		0.276	0.9
			EVI_vi_max_slope		NA; NA; 0.28	0.8

**Table 8:** Height and yield with initial (first number) and selected (in brackets) variables at the two growth periods (GPs), with the number (n.) of principal components (PCs), and related percentage (%) of variance explained. Loading values for each PC and the correlation coefficient (r) with the dependent crop trait are also specified. The variables belong to the SD metrics, as values (_vi_) of each index at 400 and 800 GDD (growing degree days). Moreover, MD metrics as daily average growth rate (avg_gr) to the day of the maximum growth rate (_maxgr) and the peak canopy.

Trait	GP	Initial (retained) variables	Retained variables	PCs (%)	Loadings (PC1; PC2; PC3)	r
Height	1	30 (1)	DVI at 400 GDD	3 (97)	−0.24	0.67
	2	82 (1)	EVI_vi_at_peak	3 (97)	0.18, 0.11	0.87

Yield	1	30 (3)	DVI_maxGR	3 (98)	NA; −0.37	0.1
			CLRE_maxGR		−0.13	0.1
			DVI AUC_maxGR		NA; −0.35; NA	0.1
	2	82 (3)	DVI_peak	3 (97)	−0.34	0.65
			CLRE_peak		−0.33	0.66
			DVI AUC_peak		−0.16; NA; 0.28	0.36

**Table 9:** Summary of the model accuracies across approaches (the single date using UAV data, SD_UAV; the multi date with UAV, MD_UAV and the multi date from PlanetScope, MD_PS) and growth periods (GPs). For each model (random forest, RF; support vector regression (SVR); gaussian process regressor (GPR); linear model (LM)) the coefficient of determination (R^2^) and the root-mean-squared error (RMSE) are specified.

Approach	GP	Predictors	Model	R^2^	RMSE (m)

SD_UAV	2	• NDRE • OSAVI	GPR	0.76	0.09
			RF	0.76	0.09
			SVR	0.77	0.08

MD_UAV	1	• EVI_avgRate_toMax • DVI_vi_max_slope	GPR	0.68	0.07
			RF	0.73	0.06
			SVR	0.75	0.06
	2	• DVI_vi_peak • NDRE • GEMI_avgRate_toPeak • EVI_avgRate_toMax • DVI_vi_max_slope • canopy_volume • x95thPercentile_height	GPR	0.86	0.05
			RF	0.86	0.04
			SVR	0.88	0.05
	3	• DVI_vi_peak • NDRE • GEMI_avgRate_toPeak • EVI_VIat_BiomassCuts • DVI_vi_max_slope • canopy_volume	GPR	0.86	0.05
			RF	0.86	0.05
			SVR	0.87	0.04

MD_PS	1	• DVI at 400 GDD	LM	0.4	0.07
	2	• EVI_vi_at_peak	LM	0.75	0.04

**Table 10:** Summary of the model accuracies across for dry matter using the SD_UAV and the MD_UAV across and growth periods (GPs). The coefficient of determination (R^2^), root-mean-squared error (RMSE) are specified for random forest, RF; support vector regression (SVR); gaussian process regressor (GPR).

Approach	GP	Predictors	Model	R^2^	RMSE (Mg/ha)

SD_UAV	2	• OSAVI • NDRE	GPR	0.74	1.54
			RF	0.72	1.58
			SVR	0.73	1.56

MD_UAV	1	• DVI_vi_max_slope • DVI_AUC_toMaxSlope	GPR	0.68	1.21
			RF	0.65	1.22
			SVR	0.73	1.08
	2	• DVI_vi_peak • NDRE • OSAVI_AUC_toPeak • DVI_vi_max_slope	GPR	0.83	0.91
			RF	0.85	0.82
			SVR	0.86	0.79
	3	• DVI_vi_peak • DVI_vi_max_slope • NDRE	GPR	0.83	0.9
			RF	0.85	0.82
			SVR	0.86	0.78

**Table 11:** Accuracies for yield predictions across three approaches for single (SD) and multi-date (MD) and growth periods (GPs) with different ML models: random forest, RF; support vector regression (SVR); gaussian process regressor (GPR); linear model (LM). The model performance is evaluated through the coefficient of determination (R^2^) and the root-mean-squared error (RMSE).

Approach	GP	Predictors	Model	R^2^	RMSE (kg/ha)

SD_UAV	2	• EVI • NDRE	GPR	0.81	361
			RF	0.80	360
			SVR	0.81	357

MD_UAV	1	• DVI_vi_max_slope • DVI_AUC_toMaxSlope	GPR	0.7	188
			RF	0.68	188
			SVR	0.72	178
	2	• DVI_vi_peak • NDRE • DVI_AUC_toPeak • DVI_vi_max_slope	GPR	0.83	141
			RF	0.82	140
			SVR	0.83	138
	3	• DVI_vi_peak • NDRE • EVI_vi_max_slope	GPR	0.83	141
			RF	0.82	139
			SVR	0.85	131

MD_PS	1	• DVI_maxGR • CLRE_maxGR • DVI AUC_maxGR	RF	Large plot: 0.48 Field://	Large plot: 670 Field://
	2	• DVI_peak • CLRE_peak • DVI AUC_peak	RF	Large plot: 0.67 Field: 0.64	Large plot: 533 Field: 1032

## Data Availability

Data are available under request from the University of Queensland.
