# Supplementary material for: From plots to commercial fields: scalable, transferable cotton morphology and productivity estimation using functional growth proxies from UAV and PlanetScope time series
Source: Plant Phenomics. 2026 May 8;8(3):100220. doi: 10.1016/j.plaphe.2026.100220 (PMC13320494; doi:10.1016/j.plaphe.2026.100220)
Supplement: Multimedia component 1 [file mmc1.docx]

# **SUPPLEMENTARY MATERIALS**

## Methodology:

### *Features from UAV orthomosaics*

To evaluate whether the buffer area and the actual cut were correlated and conveyed the same information a comparison between the two was conducted. The vegetation indices (VIs) average was derived for both the buffer area and the actual biomass cut. The buffered regions of interest (ROIs) values were averaged and used in a linear model (LM) against the average value of the VIs for the cuts. The results indicated that the two indices were not only statistically significantly correlated but also closely aligned with the 1:1 line (fitted line coefficient > 0.9) (Figure S1).


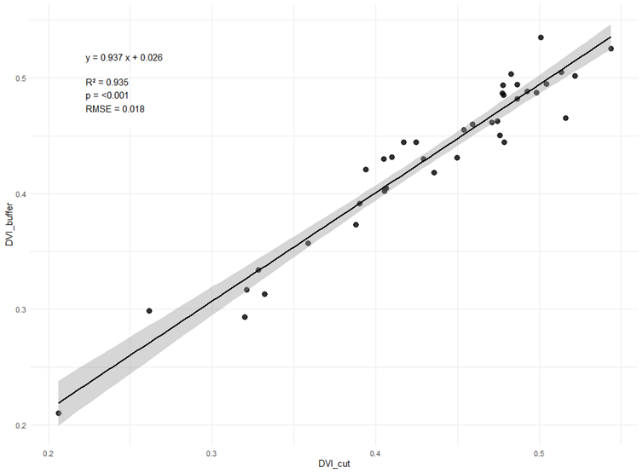

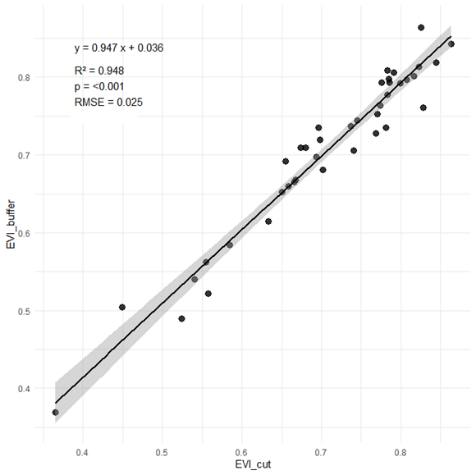

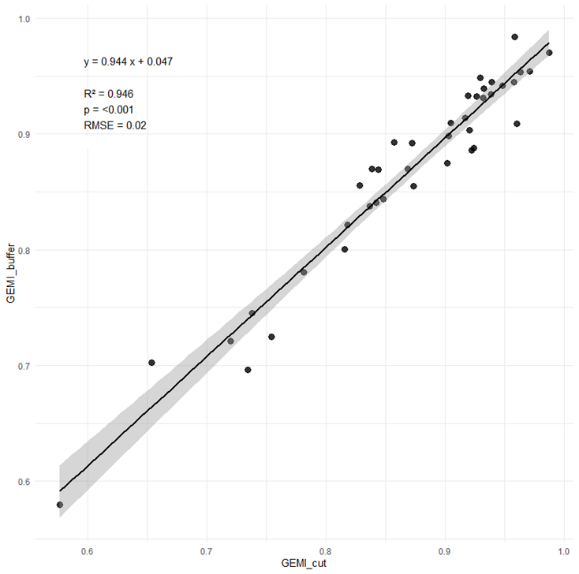

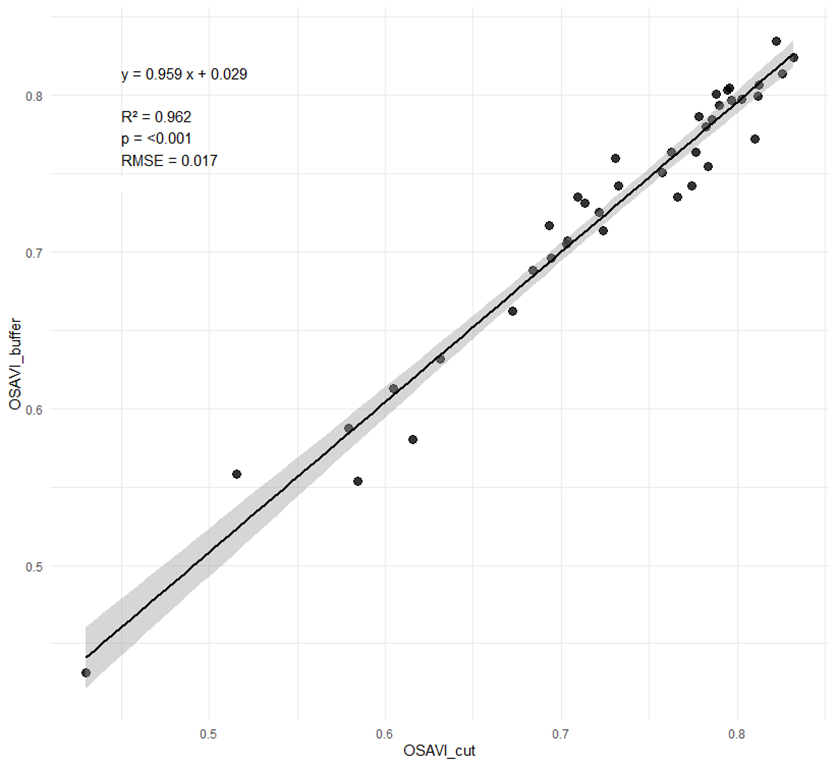


Figure S 1: Each vegetation index was derived from the actual cuts (_cut) and the buffer areas above and below the cuts (_buffer). The grey area is the 95% confidence interval (CI) band around the fitted regression line.

### *Minimising background effect from PlanetScope pixels*

The 3 m spatial resolution of the satellite imagery introduces mixed signals, where individual pixels may contain both vegetation and soil. To minimise this effect, only pixels fully enclosed within each plot boundary were retained as “pure vegetation” pixels (Figure 4). This extraction was performed in Python. Plot boundaries were reprojected to match the coordinate reference system (CRS) of the satellite data to ensure spatial alignment. To further reduce edge effects and guarantee homogeneous land cover within plots, an inward geometric buffer of 2 m was applied to each plot polygon. Spectral bands required for VIs were then extracted from these enclosed pixels, and zonal statistics were computed at the plot level.

### *PlanetScope metric extraction and adjustment for soil and cover effects*

From each plot, for the fully enclosed pixels, a set of VIs was calculated by using the same equations in Table 4. From sequential PS data using morphological indices, curve shape attributes were derived, while the biochemical features were kept as SD values from the composites at the peak canopy. To account for the bidirectional reflectance and mixed signals due to the soil-green and senescent canopy interactions all VIs were normalised (VInorm) by using the Equation 1:

$VInorm= \frac{VIi-VImin}{VImax-VImin}$ Eq. [1]

By scaling each VI value (VIi) between two endmembers (the minimum, Vimin, and maximum, VImax, values), the equation normalizes the index to estimate the proportion of vegetation, by isolating it from the soil background.

### *SG parameters for reconstructed growth curves*

To derive the growth curves, a local least-squares polynomial fit over a moving window was used. Two key parameters were calibrated for the SG’s performance:

- The half-width of the smoothing window, which controls the window size. Larger values yield smoother curves but may attenuate sharp features.
- The polynomial degree, which determines the order of the local polynomial fit, typically ranging from 2 to 4. Lower values produce smoother results at the cost of potential bias, whereas higher values reduce bias but risk overfitting and increased noise.

Given the maximum 30-day interval between images, caused by heavy rainfall, SG filter parameters were fine-tuned to a 51-point moving window centred on each data point, using a quadratic polynomial (Figure 5). This same procedure was applied for both UAV and satellite time series.

### *SHAP visualisation for feature contribution*

SHAP distributions were visualised using violin plots (Figure S2), showing both the spread and density of feature effects. Longer violins denoted features with greater and more consistent influence, while individual-coloured points, scaled by SHAP magnitude, captured variability in importance across samples.


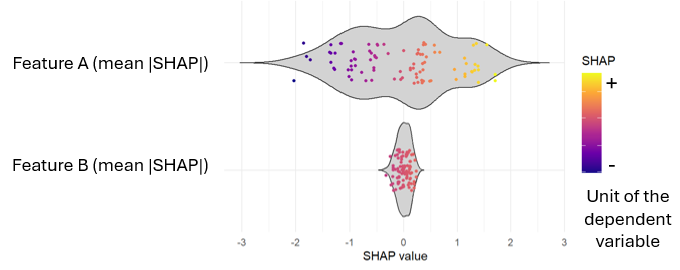


Figure S 2: SHAP contribution of features to a model prediction. Each dot represents one observation, where the horizontal position indicates the SHAP value (in the same unit as the dependent variable) and the colour scale reflects the corresponding feature value (low = purple, high = yellow). The violin shapes illustrate the distribution of SHAP values across samples. Features are in decrescent ordered by their mean|SHAP|.

### *Machine learning models for traits prediction and estimation*

The study compared three machine learning (ML) models for the UAV approaches: random forest (RF), support vector regression (SVR) and gaussian process regression (GPR). These were performed using the hyperparameter tuning with grid search method and 4-fold cross validation in RStudio (v4.4.4). RF was implemented via the ranger method while SVR and GPR were fitted using the kernlab package with a radial basis function kernel through the caret framework. Predictors were centred and scaled for SVR and GPR, whereas RF was applied to unscaled data. Table S1 provides for each algorithm the specific functions and libraries used for this approach, along with details related to the tuned hyperparameters.

Table S 1: Random Forest (RF), Support Vector Regression (SVR) and Gaussian Process Regression (GPR) model configuration, with related method and hyperparameters and details.

| **Algorithm** | **Configuration** | **Details** | **Documentation** |
| --- | --- | --- | --- |
|  | | | |
| RF | train(method = "ranger") (caret); internally uses ranger::ranger() | Nonparametric ensemble method using bagging; combines multiple decision trees for regression/classification tasks | randomForest, caret, regression |
|  | min.node.size | Minimum number of observations to split a node; controls depth and overfitting |  |
|  | ntree = 500 | Number of trees in the forest; balances variance reduction |  |
|  | splitrule = 'variance' | Splitting rule for regression; minimizes within-node variance |  |
|  | mtry | Number of predictors randomly sampled at each split; controls randomness and generalization |  |
|  | | | |
| SVR | train(method = "svmRadial") (caret); internally uses kernlab::ksvm() | SVR using RBF kernel for modeling nonlinear relationships; robust to noise and outliers | kernlab, caret, |
|  | sigma (γ) | Bandwidth parameter of RBF kernel; controls influence of training samples |  |
|  | epsilon (ε) | Error tolerance margin; ignores small deviations from actual value |  |
|  | C (cost) | Penalty parameter; balances training error vs model complexity |  |
|  | | | |
| GPR | train(method = "gaussprRadial") (caret); internally uses kernlab::gausspr() | Nonparametric kernel-based method; uses Gaussian process prior updated to posterior | Kernlab, caret |
|  | sigma | Kernel’s inverse length-scale; controls smoothness of the function |  |
